# Akkermansia muciniphila Alleviates Dextran Sulfate Sodium (DSS)-Induced Acute Colitis by NLRP3 Activation

**DOI:** 10.1128/Spectrum.00730-21

**Published:** 2021-10-06

**Authors:** Siwen Qu, Lina Fan, Yadong Qi, Chaochao Xu, Yingying Hu, Shujie Chen, Wei Liu, Weili Liu, Jianmin Si

**Affiliations:** a Department of Gastroenterology, Sir Run Run Shaw Hospital, School of Medicine, Zhejiang University, Hangzhou, China; b Department of Gastroenterology, Second Affiliated Hospital, School of Medicine, Zhejiang University, Hangzhou, China; c Department of Gastroenterology, The Second Hospital of Jiaxing, Jiaxing, China; d Institute of Gastroenterology, Zhejiang University, Hangzhou, China; e Cancer Center, Zhejiang University, Hangzhou, China; f Institute of Plant Protection and Microbiology, Zhejiang Academy of Agricultural Sciences, Hangzhou, China; Lerner Research Institute

**Keywords:** colitis, gut inflammation, inflammasome, inflammatory bowel disease, probiotics

## Abstract

Akkermansia muciniphila has been proved to play a crucial role in the progression of colitis, but its underlying mechanism remains inconclusive. In this study, we aim to investigate the effect of *A. muciniphila* on the development of acute colitis and explore the underlying mechanism. We found that the fecal level of *A. muciniphila* was decreased in ulcerative colitis (UC) patients compared to the healthy people in the GMrepo database. Oral administration of *A. muciniphila* strain BAA-835 significantly ameliorated the symptoms in dextran sulfate sodium (DSS)-induced acute colitis, evidenced by decreased body weight loss, colon length shortening, and colon histological inflammatory score. In addition, the number of goblet cells and the mucin family were enhanced after *A. muciniphila* treatment. Furthermore, proinflammatory cytokines such as tumor necrosis factor alpha (TNF-α), interleukin-6 (IL-6), and monocyte chemoattractant protein 1 (MCP-1) had a downward trend. Mechanistically, the expression of NLRP3, caspase-1 p20, and IL-1β p17 were upregulated in *A. muciniphila*-treated mice. Additionally, the colon tissues from high-*A. muciniphila* UC patients had a higher NLRP3 expression than that from low-*A. muciniphila* UC patients. Moreover, the upregulation of NLRP3 was observed in mouse macrophage Raw264.7 cells and bone marrow-derived macrophage (BMDM) cells after incubation with *A. muciniphila*. To clarify whether the protective effect of *A. muciniphila* in colitis depends on NLRP3, we performed the NLRP3-deficient assay in NLRP3^−/−^ mice *in vivo*. The evidence showed that NLRP3 deficiency eliminated the protective effects of *A. muciniphila* in acute colitis. In conclusion, *A. muciniphila* alleviates DSS-induced acute colitis by NLRP3 activation, which enriches the mechanism and provides a new prospect for the probiotic-based treatment of colitis.

**IMPORTANCE** The gut microbiota and host immune response interaction influences the progression of intestinal inflammatory disease. As a well-recognized next-generation probiotic, Akkermansia muciniphila has been proved to play a crucial role in the progression of colitis, but its underlying mechanism remains inconclusive. We found that oral administration of *A. muciniphila* strain BAA-835 significantly ameliorated the symptoms of acute colitis. Mechanistically, the expression of NLRP3 was upregulated in the *A. muciniphila* group, and the protective effect of *A. muciniphila* in colitis depends on NLRP3 activation. This enriches the mechanism and provides a new prospect for the probiotic-based treatment of colitis, which would promote a deeper understanding of the complex characteristics of *A. muciniphila* and provide guidance for the treatment of human colitis in the future.

## INTRODUCTION

The development of inflammatory bowel disease (IBD) is affected by numerous complicated elements (1). Except for hereditary characteristic and environmental factors, the gut microbiota is a new key contributor (2, 3). IBD encompasses ulcerative colitis (UC) and Crohn’s disease (CD). UC is associated with the gut dysbiosis following compositional and metabolic changes of intestinal flora (3, 4). Gut microbiota disturbance leads to the increase of harmful bacteria and results in gut homeostasis imbalance (4). Probiotics can build up a healthy ecosystem and prevent the host from experiencing pathogenic infections (5, 6). Representative probiotics such as *Lactobacillus* and *Bifidobacterium* are widely proved to be beneficial to maintain gut barrier function, modulate local immune response, and enhance digestion (7–9). Many probiotics have been developed as live biotherapeutic products (LBPs) in clinical diseases (10).

Akkermansia muciniphila, a Gram-negative and strictly anaerobic bacterium, was first isolated from human faces in 2004 (11). It belongs to the *Verrucomicrobia* phylum, which was found to be abundant in the human gut (12, 13). *A. muciniphila* has an inverse correlation with some metabolic diseases, such as overweight, obesity, and type 2 diabetes disease (14, 15). *A. muciniphila* supplementation in mice or humans shows good efficacy, safety, and tolerance (16). Moreover, studies reveal that *A. muciniphila* could protect against serious diseases such as atherosclerosis (17), ALS (amyotrophic lateral sclerosis) (18), and immune-mediated liver injury (19). Transplantation with *A. muciniphila* increased the survival percentage in progeric mice (20). It exerts a beneficial effect by improving the gut barrier and regulating bile acid metabolism (15, 17, 19). *A. muciniphila* has been widely regarded as a promising candidate of next-generation probiotics (NGP) (21, 22). In recent years, *A. muciniphila* has been proved to be related to human and murine colitis. However, its exact function and underlying mechanism have not been explored.

The purpose of this study is to confirm the effect of *A. muciniphila* on colitis, including the impact on symptom phenotype as well as its potential molecular mechanism. It will promote a deeper understanding of the complex characteristics of *A. muciniphila* and provide guidance for the treatment of human colitis in the future.

## RESULTS

### The abundance of *A. muciniphila* is decreased in UC patients.

**FIG 1:**
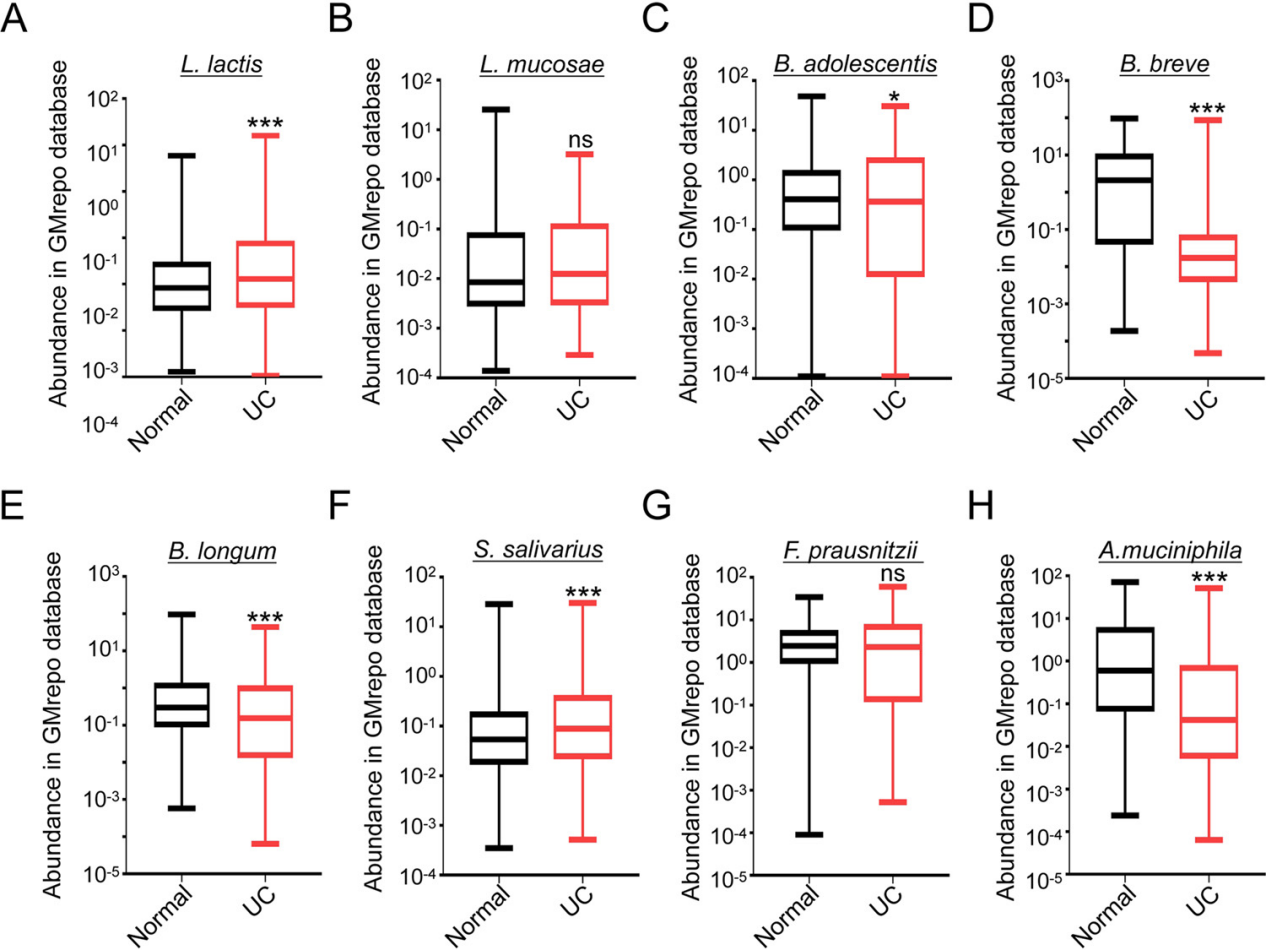
The abundance of *A. muciniphila* is decreased in UC patients. (A to H) The relative fecal abundance of L. lactis (normal = 1,000, UC = 480), *L. mucosae* (normal = 549, UC = 43), *B. adolescentis* (normal = 1,000, UC = 473), *B. breve* (normal = 1,000, UC = 317), B. longum (normal = 1,000, UC = 658), *S. salivarius* (normal = 1,000, UC = 1,000), *F. prausnitzii* (normal = 1,000, UC = 1,000), and *A. muciniphila* (normal = 1,000, UC = 420) in the stool of normal people and UC patients were analyzed in the GMrepo database. *, *P* < 0.05; ***, *P* < 0.001; ns, no significance (Mann-Whitney test); UC, ulcerative colitis.

First, we analyzed the well-known probiotic levels of Lactobacillus lactis, Lactobacillus mucosae, Bifidobacterium adolescentis, Bifidobacterium breve, Bifidobacterium longum, Streptococcus salivarius, Faecalibacterium prausnitzii, and *A. muciniphila* in the stool of healthy individuals and UC patients in the GMrepo database. Then we found the reduced levels of *B. adolescentis*, *B. breve*, B. longum, and *A. muciniphila* in the UC patients compared to the healthy people, but the levels of L. lactis and *S. salivarius* were increased in UC patients, while *L. mucosae* and *F. prausnitzii* had no difference (Fig. 1A to H). In addition, the GMrepo database showed that the fecal abundance of *A. muciniphila* was remarkably reduced in IBD patients compared to the healthy people (see Fig. S1A in the supplemental material). The details of bacteria, including exact sample numbers, medians, and values are shown in Fig. S1B. These data indicate that there might be a relationship between *A. muciniphila* and clinical colitis.

### *A. muciniphila* alleviates the phenotype in DSS-induced acute colitis.

**FIG 2:**
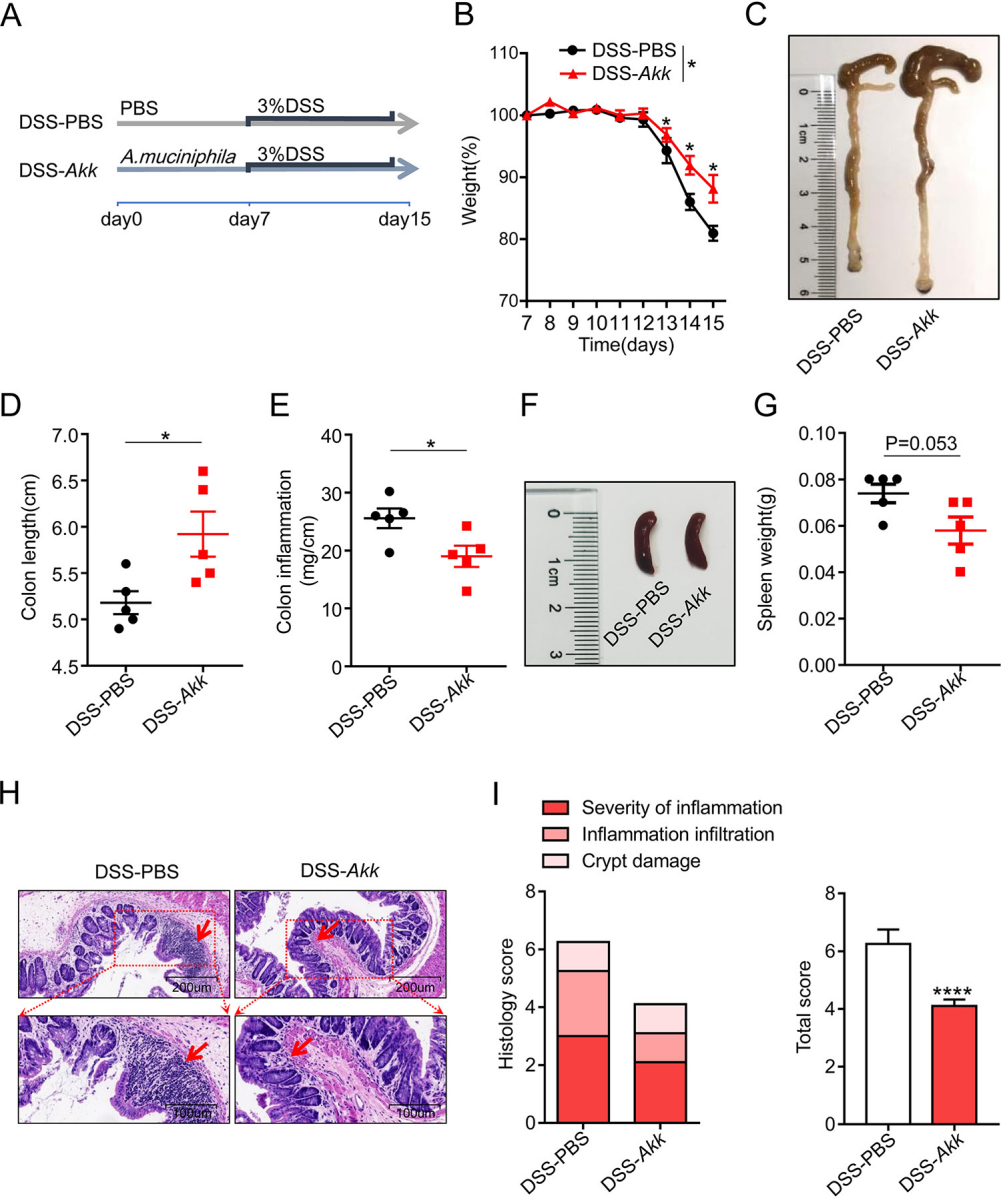
*A. muciniphila* alleviates the phenotype in DSS-induced acute colitis. (A) Experiment design of the acute DSS-induced colitis model. (B) Mouse weight loss was recorded daily. (C to E) PBS- and *A. muciniphila*-gavaged mice were sacrificed at day 15, and then the representative colons were photographed. Then the lengths and weights of the colons were measured, and colon inflammation was calculated by dividing the colon weight by the colon length. (F to G) Spleens were harvest and weighed. The representative spleens were photographed. (H) Representative pictures of H&E-stained colon tissue. (I) The histology score was the sum of inflammation damage, inflammation infiltration, and crypt damage scores. Data are expressed as the mean ± SD; *n* = 5 per group. *, *P* < 0.05; ****, *P* < 0.0001 (unpaired Student’s *t* test). Red arrows, inflammatory cells; *Akk*, Akkermansia muciniphila.

Based on the GMrepo database results, we hypothesized that *A. muciniphila* is associated with the occurrence and development of colitis. Then we built an acute colitis model to verify the effect of *A. muciniphila* on colitis. First, mice were fed with 2 mg/ml streptomycin in the drinking water for 3 days to ensure the consistency of regular microbiota. Then mice were gavage-fed phosphate-buffered saline (PBS) control or *A. muciniphila* for 7 days, followed by 3% DSS treatment for 8 days to induce colitis (Fig. 2A). We found that mice gavage-fed *A. muciniphila* (DSS-*Akk* group) showed less weight loss than the PBS-control group (DSS-PBS group) (Fig. 2B). The colon shortening and the intestinal inflammation (colon weight-to-length ratio) were decreased in the DSS-*Akk* group compared to the DSS-PBS group (Fig. 2C to E). The same trend was observed for spleen weight (Fig. 2F and G). Moreover, the histological examination of colon hematoxylin and eosin (H&E) staining showed the decreased histological score in the *A. muciniphila* group evidenced by inflammation severity, inflammation infiltration, and crypt damage (Fig. 2H and I). These results suggest that *A. muciniphila* refines the phenotype of DSS-induced acute colitis.

### NLRP3 activation involved in the protective effect of *A. muciniphila* on colitis.

**FIG 3:**
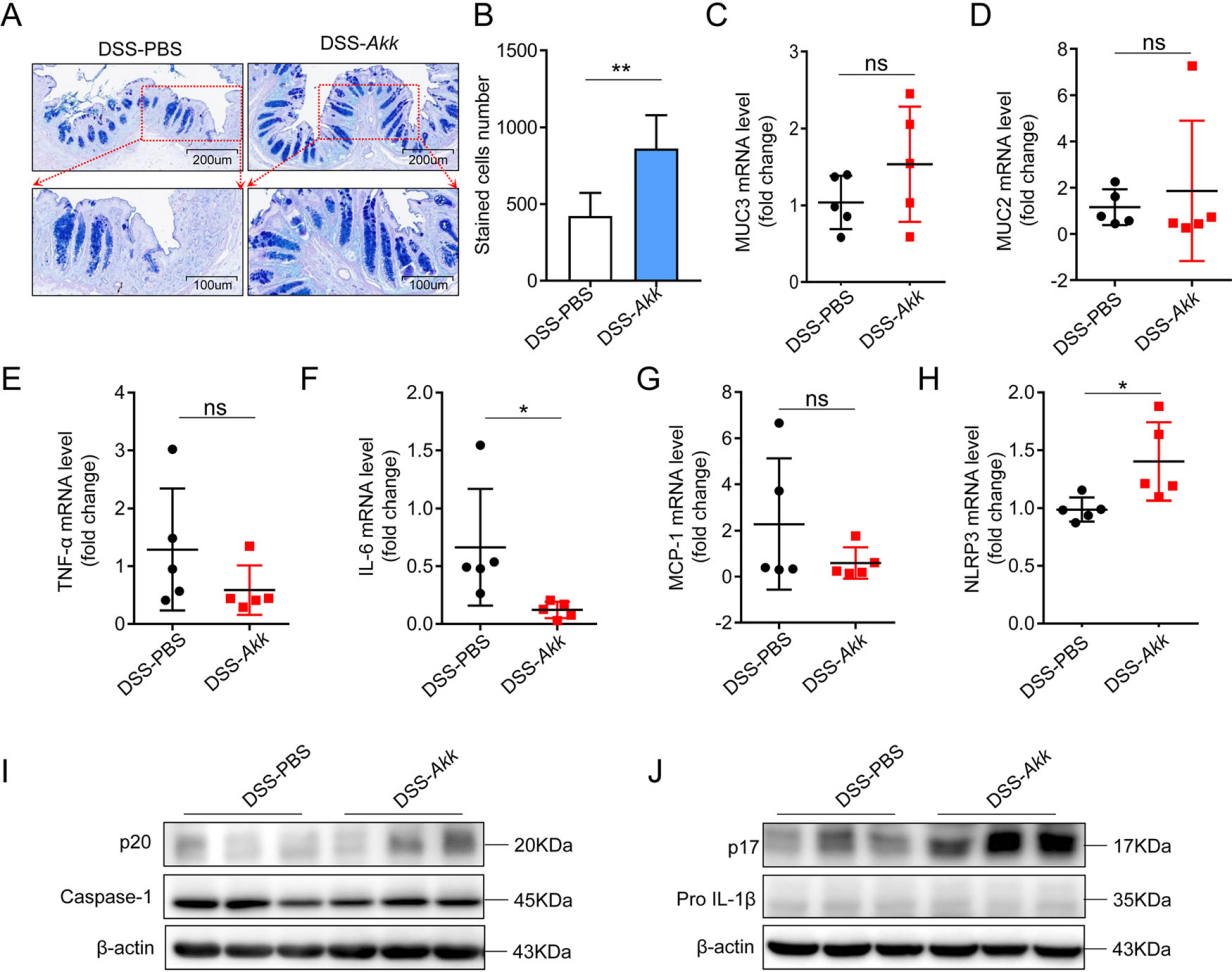
NLRP3 activation involves in the protective effect of *A. muciniphila* on colitis. (A and B) Representative pictures of alcian blue- and PAS-stained colon tissues in the PBS and *A. muciniphila* groups with DSS treatment. (C and D) The relative mRNA expression levels of *MUC2* and *MUC3* in the mice with or without *A. muciniphila* treatment were examined by q-PCR. (E to G) The relative mRNA expression levels of TNF-α, MCP-1, and IL-6 in the DSS mouse colon tissues were tested by q-PCR. (H) The relative mRNA expression levels of *NLRP3* in the mouse colon tissue were examined by q-PCR. *n* = 5 per group. (I and J) The relative expression levels of casepase-1, casepase-1 p20, IL-1β, and IL-1β p17 were measured by Western blotting. Data are expressed as the mean ± SD; *n* = 3 per group. *, *P* < 0.05; **, *P* < 0.01 (unpaired Student’s *t* test). *Akk*, Akkermansia muciniphila.

To investigate the effect of *A. muciniphila* on the colon epithelium barrier in DSS-induced colitis, we investigated the density of goblet cells using alcian blue and periodic acid-Schiff (PAS) staining. Consistent with the phenotype, we found that *A. muciniphila* increased the abundance of goblet cells compared to the control group (Fig. 3A and B). It is known that mucin is a critical component that is made of the gut barrier (23), so we investigated the expression level of muc2 and muc3 in this model. The expression level of *Muc2* and *Muc3* had an increase trend in the *A. muciniphila* group compared to the control group (Fig. 3C and D). To identify whether *A. muciniphila* influenced the inflammatory response in the colon tissue, we examined the mRNA level of tumor necrosis factor alpha (TNF-α), interleukin-6 (IL-6), and monocyte chemoattractant protein 1 (MCP-1) and then we found a decreased trend in the *A. muciniphila* group colon tissues compared with the PBS group (Fig. 3E to G). These results show that *A. muciniphila*-induced gut barrier reinforcement and inflammatory cytokine arrest contributes to the restoration of colitis.

According to the results described above, *A. muciniphila* has a protective effect on murine colitis, but the underlying mechanism has not been illustrated. There have been many studies of the correlation between NLRP3 and colitis (24). One study reported that NLRP3 is an important central regulator of pathogen recognition, host immunity, and inflammation, with utmost importance in human diseases (25). We observed the upregulated mRNA and protein expression of *NLRP3* in the mouse colon tissue treated with *A. muciniphila* (Fig. 3H). It is known that NLRP3 mediates the activation of caspase-1 and the subsequent release of mature IL-1β (26). Therefore, we evaluated the protein expression of NLRP3 downstream molecules, caspase-1 p20 and mature IL-1β p17. We observed that caspase-1 p20 and IL-1β p17 had an upregulated trend after *A. muciniphila* treatment (Fig. 3I and J). These data indicated that NLRP3 activation involves in the protective effect of *A. muciniphila* on colitis.

### *A. muciniphila* abundance correlates with the expression of NLRP3 in colitis.

**FIG 4:**
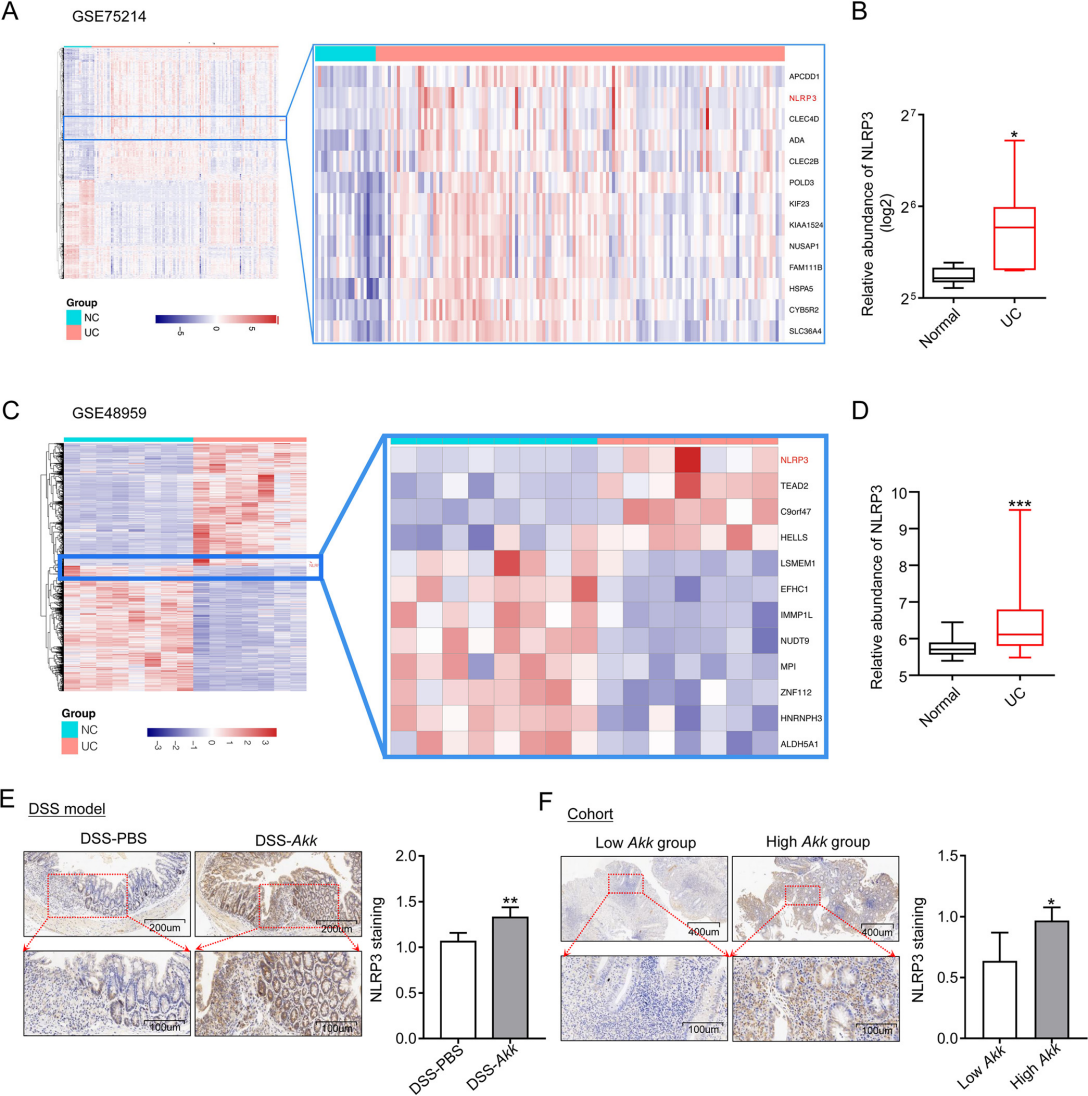
*A. muciniphila* abundance correlates with the expression of NLRP3 in colitis. (A and C) Heatmap hierarchically demonstrated DEGs in the GSE75214 and GSE48959 ulcerative colitis group compared with normal patients. Red and blue indicate higher expression and lower expression, respectively (GSE75214: NC = 20, UC = 135; GSE48959: NC = 8, UC = 7). (B and D) The relative expression of NLRP3 in the GSE75214 and GSE48959 cohorts. (E) Representative abundances of NLRP3 were tested in the colon tissues from PBS- and *A. muciniphila*-gavaged mice with DSS treatment by immunohistochemical staining; *n* = 5 per group. (F) The UC patients were divided into low- and high-*A. muciniphila* groups according to the tissue abundance of *A. muciniphila* (-Δ*^CT^*), and then the abundances of NLRP3 from the low-*A. muciniphila* and high-*A. muciniphila* groups were explored by immunohistochemical staining; *n* = 6 per group. Data are expressed as the mean ± SD. *, *P* < 0.05; **, *P* < 0.01; ***, *P* < 0.001 (Mann-Whitney test or unpaired Student’s *t* test).

In combination with NLRP3 activation, we wondered if there is relevance among *A. muciniphila*, NLRP3, and colitis. First, we investigated the relationship between NLRP3 and human UC. The differentially expressed genes (DEGs) from two Gene Expression Omnibus (GEO) cohorts (RNA-seq from the healthy and ulcerative colitis patients; GEO number GSE75214; normal control [NC] = 20, UC = 135; GEO number GSE48959; NC = 8, UC = 7) were calculated and plotted as a hierarchical-clustering heat map. The NLRP3 gene was listed as one of the significant upregulated genes (Fig. 4A and C). Moreover, we extracted boxplots to validate the expression level of NLRP3 in the UC patients and normal controls (Fig. 4B and D).

To further verify the relationship between *A. muciniphila* and NLRP3 expression in colitis, we tested the expression of NLRP3 in the DSS-induced colitis model. Immunohistochemistry staining showed that the mice treated with *A. muciniphila* had a higher NLRP3 expression than the PBS control group (Fig. 4E). In addition, we divided the UC patients into two groups, low-*A. muciniphila* and high-*A. muciniphila*, according to the tissue abundance of *A. muciniphila*. Patient characteristics included gender, age, body size, and tobacco and alcohol consumption were calculated (Table S1) to ensure the comparability of the two sets of data. The high-*A. muciniphila* group had higher NLPR3 expression than the low-*A. muciniphila* group, as determined by immunohistochemistry (Fig. 4F). These results suggest that NLRP3 is upregulated in the UC patients, and there is a positive correlation between *A. muciniphila* abundance and NLRP3 expression in colitis.

### *A. muciniphila* increases the expression of NLRP3 *in vitro*.

**FIG 5:**
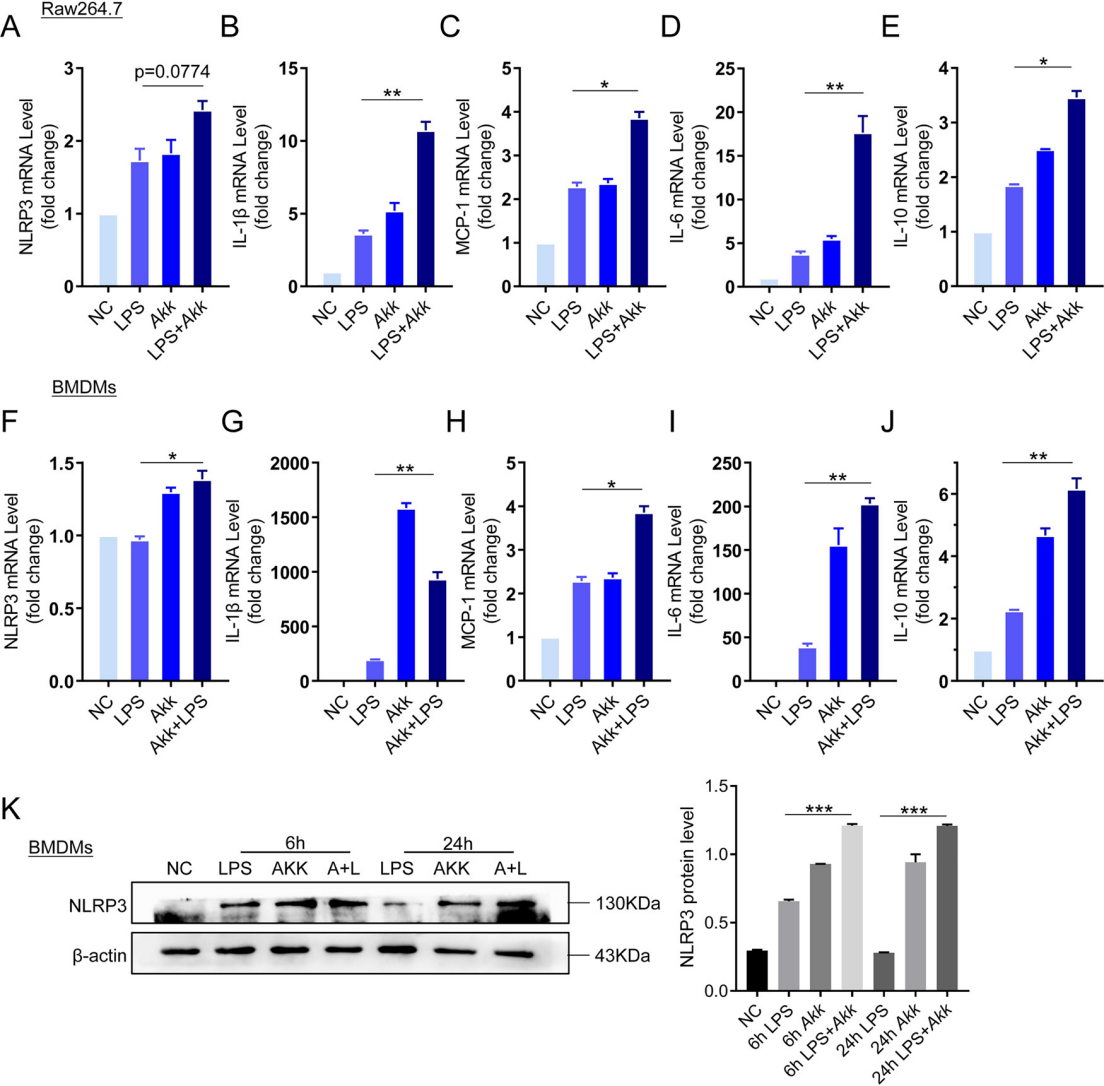
*A. muciniphila* increases the expression of NLRP3 *in vitro*. (A to J) Raw264.7 cells and BMDMs were incubated with LPS or *A. muciniphila*, and then the mRNA expression of NLRP3, IL-1β, MCP-1, IL-6, and IL-10 in Raw264.7 cells and BMDMs was tested by q-PCR. (K) BMDMs were cocultured with LPS or *A. muciniphila* for 6 h or 24 h, and then the expression of NLRP3 was examined by Western blot analysis, and the quantitative graph was shown. Data are expressed as the mean ± SD; *n* = 3 per group. *, *P* < 0.05; **, *P* < 0.01; ***, *P* < 0.001 (ANOVA test). NC, negative control; *Akk*, Akkermansia muciniphila; LPS, lipopolysaccharide.

NLRP3 has been proved to be associated with *A. muciniphila* abundance in colitis in both mice and humans. We wondered whether *A. muciniphila* promoted NLRP3 expression *in vitro*. In the active phase of UC disease, NLRP3 was expressed in the immune cells in the lamina propria and was absent from the epithelial cells (27). Therefore, we used mouse macrophage cell line Raw264.7 cells and mouse bone marrow-derived macrophages (BMDMs) to evaluate the effect of *A. muciniphila* on NLRP3 activation and proinflammatory cytokine production. In Raw264.7 cells, results showed that the mRNA expression level of NLRP3, IL-1β, MCP-1, IL-6, and IL-10 were significantly increased after cells were treated with lipopolysaccharide (LPS) for 12 h (500 ng/ml) (Fig. 5A to E). After being cocultured simultaneously with *A. muciniphila* (100:1) and LPS (500 ng/ml), NLRP3, IL-1β, MCP-1, IL-10 and IL-6 were remarkably increased compare to the LPS-alone group. Similar results were confirmed in BMDM cells (Fig. 5F to J). Moreover, we found that the protein level of NLRP3 was significantly increased after LPS incubation along with *A. muciniphila* compared to that of the LPS-alone group at the 6-h and 24-h time points (Fig. 5K). These results indicate that *A. muciniphila* induces the expression of NLRP3 and proinflammatory cytokines *in vitro*.

### NLRP3 deficiency eliminates the protective effect of *A. muciniphila* in colitis.

**FIG 6:**
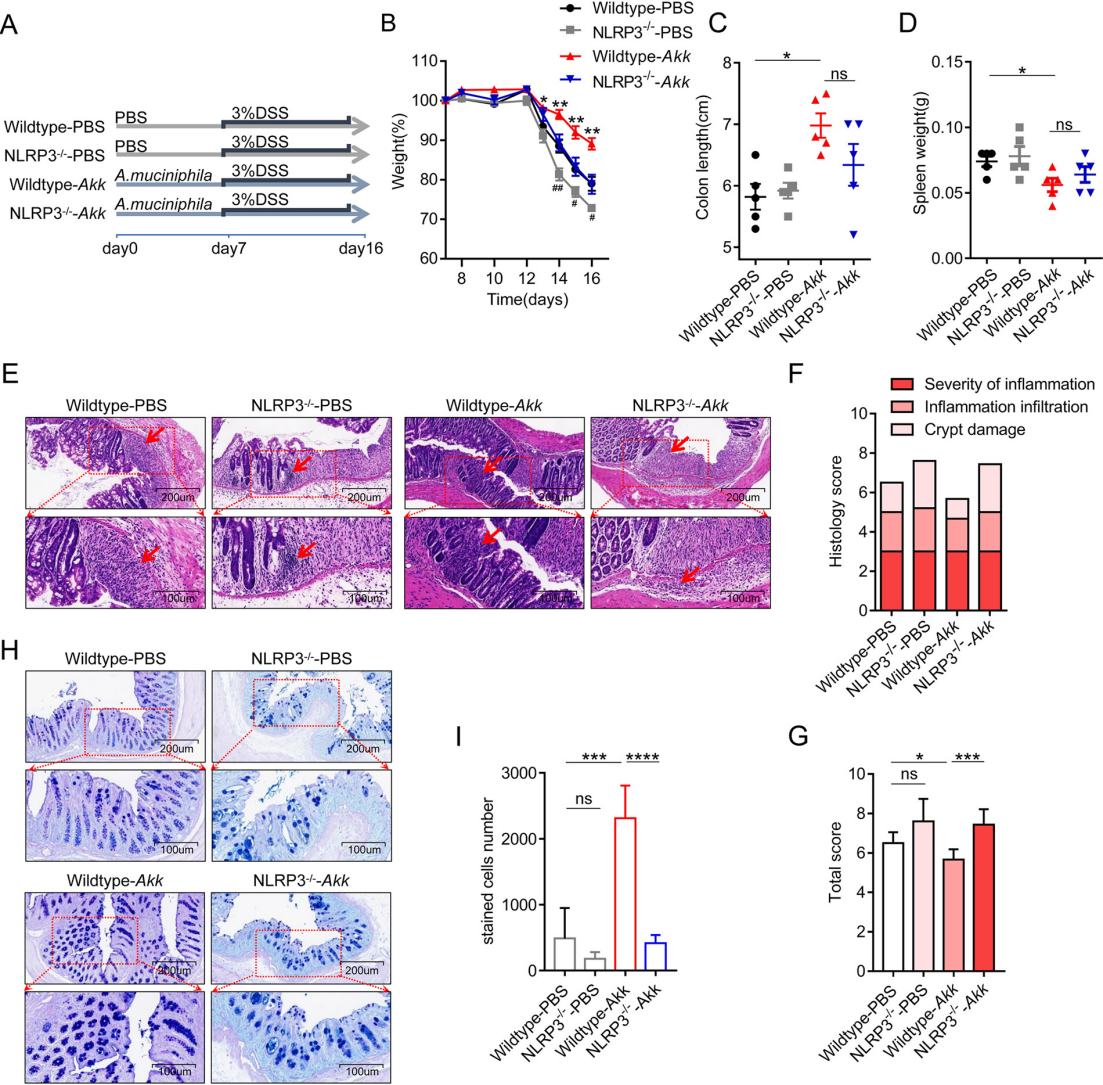
NLRP3 deficiency eliminates the protective effect of *A. muciniphila* in colitis. (A) Experiment design of the mouse model for acute DSS-induced colitis in NLRP3^−/−^ mice. (B) Weight loss in the wild-type or NLRP3^−/−^ mice with or without *A. muciniphila* treatment. (*, wild-type-*A. muciniphila* compared to wild-type-PBS; #, NLRP3^−/−^-PBS compared to wild-type-PBS). (C and D) Mice were sacrificed on day 16, and the colon length and spleen weight were measured. (E to G) Representative pictures of H&E-stained mouse colon tissue in the wild-type and NLRP3^−/−^ mice with or without *A. muciniphila* treatment. The histology score was the sum of the inflammation damage, inflammation infiltration, and crypt damage scores. (H and I) Representative pictures of alcian blue- and PAS-stained colon tissue in the wild-type and NLRP3^−/−^ mice with or without *A. muciniphila* treatment. Red arrows, inflammation cells. Data are expressed as the mean ± SD or standard error of the mean (SEM); *n* = 5 per group. *, *P* < 0.05; **, *P* < 0.01; ***, *P* < 0.001; ****, *P* < 0.0001; ns, no significance (ANOVA test). *Akk*, Akkermansia muciniphila.

To clarify whether the protective effect of *A. muciniphila* in colitis depends on NLRP3, we performed the NLRP3-deficent assay. We gavage-fed the wild-type (WT) and NLRP3 knockout mice (*NLRP3^−/−^*) with or without *A. muciniphila* treatment with DSS application (Fig. 6A). We observed that mice supplemented with *A. muciniphila* alleviated the colitis symptoms as described by less weight loss, colon length shortening, and spleen weight. As expected, the NLRP3^−/−^ mice gavage-fed *A. muciniphila* showed an increased trend compared to the wild-type mice with *A. muciniphila* treatment in weight loss, colon length shortening, and spleen weight (Fig. 6B to D). Consistent with the symptoms, when comparing the *A. muciniphila* group with the PBS-control group, we found less extensive destruction of villous epithelium, disappearance of crypts, and infiltration of inflammatory cells in the *A. muciniphila* group using H&E staining (Fig. 6E and F). Meanwhile, NLRP3^−/−^ mice gavage-fed *A. muciniphila* increased the histological score compared with the wild-type mice gavage-fed *A. muciniphila* (Fig. 6G). In addition, we found the same trend with alcian blue and PAS staining (Fig. 6H and I). Taken together, these results suggest that the therapeutic effect of *A. muciniphila* in colitis depends on NLRP3.

## DISCUSSION

The gut microenvironment function in the progression of UC is gradually being explored. The DSS-induced colitis model is an economical and easily manipulated option which induces colon epithelial injury and gut immune response. Thus, it is widely applied in studies of the interaction between human IBD and gut microbiota (28–31). Studies have reported *A. muciniphila* to be decreased in UC patients (2), which is consistent with our findings from the GMrepo database results (Fig. 1). However, it is still unknown how *A. muciniphila* decreases in UC patients, and this needs further exploration.

In this study, we found that *A. muciniphila* supplementation significantly relieved the symptoms in mice with acute colitis (Fig. 2). *A. muciniphila* was considered to renew the mucus layer and maintain gut integrity (32). In our experiment, we found that *A. muciniphila* supplementation reduced the inflammatory cell infiltration and increased the number of goblet cells and expression of *MUC2* and *MUC3* (Fig. 3). A previous study also found that *A. muciniphila* administration ameliorates DSS-induced ulcerative colitis by enhancing gut barrier function (31). Gut inflammation is accompanied by infiltration of immune cells and goblet cell impairment (1, 33). The mucin family reflects the function of the gut barrier and protects the epithelium from harmful factors (34). Moreover, *A. muciniphila* decreased the expression of proinflammatory cytokines of IL1β, MCP-1, and IL-6, which were secreted by local activated inflammatory cells (Fig. 3). These results indicated that *A. muciniphila* has a preventive effect on experimental colitis.

The effect of *A. muciniphila* on colitis is controversial in some studies. Studies stated that *A. muciniphila* exacerbates gut inflammation in Salmonella enterica serovar Typhimurium-infected mice (35). NLRP6 deficiency aggravates intestinal inflammation in IL-10^−/−^ mice by the upregulation of *A. muciniphila* (36). However, there is no evidence that *A. muciniphila* alone causes pathogenicity (37). *A. muciniphila* alone cannot promote intestinal inflammation and cause pathogenicity in IL-10^−/−^ mice. In the meanwhile, its metabolites mucin degradants are considered beneficial to the regulation of the host immune system (11, 38). *A. muciniphila* colonizes the superficial mucosa and maintains intestinal microbial homeostasis by competing with harmful mucus-degrading bacteria (39). Interactions with specific microbes or molecules from other commensals might change the function of *A. muciniphila* (40).

Our study demonstrated that *A. muciniphila* supplementation leads to NLRP3 upregulation *in vitro* and *in vivo* (Fig. 4 and 5). Moreover, NLRP3 deficiency negated the protective effect of *A. muciniphila* in colitis (Fig. 6). NLRP3 belongs to the nucleotide oligomerization domain (NOD)-like receptor family, which is responsible for auto-inflammatory disorders (41). NLRP3 combining with ASC forms an inflammasome and then activates caspase-1 to cleave pro-IL-1β into its mature form (42). NLRP3 activation induces IL-1β and IL-18 release, which are acknowledged to defend against pathogens and external stimuli (33, 43). Deficiency of NLRP3 increases susceptibility to experimental colitis in mice (44–46), and it is rescued by exogenous IL-1β or IL-18 (43). In addition, hyperactive NLRP3 maintains gut homeostasis by inducing Treg cells (47). Therefore, we speculate that the expression of NLRP3 might be related to the colon epithelial repair in gut inflammation. In our study, NLRP3 was significantly upregulated in colon tissues of the UC patients, which can be considered a protective evaluation. These data suggest that NLRP3 plays a protective role in the probiotic-based therapy of colitis. However, the specific mechanism of its regulation deserves further exploration.

In conclusion, our study confirmed the protective effect of *A. muciniphila* on acute colitis and explored the possibility that this effect depends on NLRP3 activation. Our study suggests that regular *A. muciniphila* treatment might improve the therapeutic effect for inflammatory bowel disease.

## MATERIALS AND METHODS

### GMrepo database analysis.

GMrepo RESTful APIs for R version 3.6.1 and RStudio version 1.1.442 were used to obtain microbiota relative abundances in the stool samples of the healthy and UC patients from the GMrepo database. First, we assessed the data quality by consulting the description of the samples and clinical information. Finally, the relative abundance of L. lactis, *L. mucosae*, *B. adolescentis*, *B. breve*, B. longum, *S. salivarius*, *F. prausnitzii*, and *A. muciniphila* for the healthy and UC patients were extracted and analyzed.

### Human sample collection.

Fresh colon tissues were obtained from 12 UC patients who underwent colonoscopy examination at Sir Run Run Shaw Hospital, Zhejiang University School of Medicine (Zhejiang, China). All of the patients were first pathologically diagnosed and were free off anti-inflammatory or immunosuppressive drugs. The clinical characteristics of the patients are presented in Table S1. All samples were coded in accordance with local ethical guidelines (as stipulated by the Declaration of Helsinki), and written informed consent was obtained. This study was approved by the Clinical Research Ethics Committee of the Sir Run Run Shaw Hospital School of Medicine at Zhejiang University.

### DNA extraction and bacterial quantification.

Stool kits (catalog [cat.] no. 51604; Qiagen, Germany) were used for bacterial DNA extraction from human stool samples. Qiagen DNA minikits (cat. no. 56304) were used for DNA extraction from colon tissues according to the manufacturer’s protocol. Bacterial quantification was measured by quantitative real-time PCR in a Roche LightCycler 480 system (Rotor Gene 6000 software; Sydney, Australia). Each reaction was performed in triplicate with SYBR Premix *ex taq* (RR820A; TaKaRa, Japan), primers, and template DNA. Universal eubacteria 16S was used as the internal reference gene for stool samples. The PGT gene was used as the internal control for tissue samples. The following primer sets were used: *A. muciniphila* 5′-CAGCACGTGAAGGTGGGGAC-3″ (forward) and 5′-CCTTGCGGTTGGCTTCAGAT-3″; universal eubacteria 16S 5′-CGGCAACGAGCGCAACCC-3″ (forward) and 5′-CCATTGTAGCACGTGTGTAGCC-3′ (reverse); PGT 5′-ATCCCCAAAGCACCTGGTTT-3″ (forward) and 5′-AGAGGCCAAGATAGTCCTGGTAA-3′ (reverse).

### Bacterial strain and growth conditions.

*A. muciniphila* was purchased from the American Type Culture Collection (ATCC; *A. muciniphila* BAA-835). *A. muciniphila* BAA-835 lyophilized powder was inoculated in brain heart infusion (BHI) broth (BD Difco, Sparks, MD, USA) supplemented with 0.05% mucin type II (Sigma-Aldrich, Northbrook, IL) and cultured under anaerobic conditions of 10% H_2_, 10% CO_2_, and 80% N_2_ (AW500SG anaerobic workstations; Electrotek, England) at 37°C. Monoclonal floras were harvested 3 days later and continued to amplification. 16S ribosomal sequencing (V4 sequences) was performed to confirm bacterial strains at the species level. Bacteria were preserved at –80°C with 20% glycerol.

### Animal models.

Male C57BL/6 mice (6 to ∼8 weeks old) were bought from the Shanghai Laboratory Animal Center (SLAC), China. First, mice were fed 2 mg/ml streptomycin in the drinking water for 3 days to ensure the consistency of regular microbiota, and then they were randomly assigned to two groups, the *A. muciniphila* group and the PBS group. The *A. muciniphila* group was gavage-fed *A. muciniphila* (1 × 10^9^ CFU) in 300 μl PBS solution, while the PBS group was treated with 300 μl PBS for 7 days. Then the mice were administered 3% dextran sulfate sodium salt (DSS; 0216011080, MP Biomedicals) in daily drinking for 8 days to induce the colitis. Mice were weighed every other day. After the mice were sacrificed, colon tissues were weighed and the length was measured, and spleen tissues were weighed.

For the NLRP3 knockout mouse model, male C57BL/6J NLRP3^tm1Bhk^ (NLRP3^−/−^) mice were purchased from Jackson Laboratory (Maine, USA) and kept in specific-pathogen-free conditions. All animal experimental procedures were approved by the ethics committee. All animal studies were performed in accordance with the guidelines of the Institutional Animal Use and Animal Experimentation Ethics Committee at Zhejiang University. All mice were maintained in ventilated cages with 12-h light/dark cycles, enriched water, and *ad libitum* feeding under specific-pathogen-free (SPF) conditions.

### Assessment of histological score.

Colon tissues were settled as a ring and fixed overnight with 10% formalin at room temperature and then embedded in paraffin. Sections were stained with H&E for pathological analysis. Two investigators who were blinded to the treatment evaluated the slides independently. A 0- to 4-point scale was used to describe the severity of inflammation (0 = none, 1 = mild, 2 = moderate, and 3 = severe), the level of inflammation involvement (0 = none, 1 = mucosa, 2 = mucosa and submucosa, and 3 = transmural), and the extent of epithelial/crypt damage (0 = none, 1 = basal 1/3, 2 = basal 2/3, 3 = crypt loss, 4 = crypt and surface epithelial destruction). Each parameter was calculated and summed to obtain the overall score.

### Microarray expression data information.

The data sets (GSE48959 and GSE75214) were obtained from the Gene Expression Omnibus (GEO; https://www.ncbi.nlm.nih.gov/geo/) of NCBI using the R version 3.7 and GEO query packages. We retrieved the expression matrix and calculated differentially expressed genes (DEGs) from these two GEO gene-expression-profiling data set, which contained the healthy and ulcerative colitis patients. Briefly, microarray data were normalized if necessary, and the probe was further annotated to the gene by corresponding transformation. The DEGs were calculated using the limma R package. DEGs with an adjusted *P* value of <0.01 and |log2FC| of ≥2 were shown as heatmaps by heatmap R packages. The relative mRNA expression of the NLRP3 gene in the two cohorts was obtained from a normalized matrix and shown as a boxplot.

### Cell culture.

Raw264.7 was obtained from the American Type Culture Collection (ATCC; Manassas, VA, USA). It was cultured in RPMI 1640 medium (Genom, China) containing 10% fetal bovine serum (FBS) and 1% streptomycin and penicillin. The bone marrow cavities of the legs were opened and then repeatedly flushed with Dulbecco’s modified or Eagle’s medium (DMEM; Gibco) supplemented with 10% FBS (Gibco) and 2% penicillin/streptomycin. The cells were cultured in complete DMEM with mouse macrophage colony-stimulating factor (M-CSF; 20 ng/ml; Novoprotein, China). Six days later, BMDMs were confirmed by flow cytometry for F4/80 (565411, Clone T45-2342; 1 μg/ml; BD Biosciences). Differentiated macrophages (BMDMs) were collected for further experiments. All cell lines were maintained at 37°C in a humidified 5% CO_2_ atmosphere.

### RNA extraction and quantitative real-time PCR.

RNA was extracted from macrophages or fresh frozen colon tissues using TRIzol reagent (TaKaRa, Japan) according to the operation instructions. The reverse transcription real-time PCR was performed using a PrimeScript real-time (RT) reagent kit (TaKaRa). Quantitative real-time PCR was performed using SYBR premix *ex taq* (TaKaRa) in the LightCycler 480 real-time PCR system (Roche) using cDNA. Each reaction was assayed in triplicate in 10-μl reactions and was assessed using the comparative cycle method (2^−ΔΔ^*^CT^*). The results were normalized to the expression of GAPDH (glyceraldehyde-3-phosphate dehydrogenase). The final results of the operating groups were calculated relative to the control group. The mRNA expressions of mouse genes were analyzed with the following specific primers listed (GAPDH served as the internal reference gene): TNF-α 5′-CGTGCTCCTCACCCACAC-3″ (forward) and 5′-GGGTTCATACCAGGGTTTGA-3″ (reverse); NLRP3 5′-AGAGCCTACAGTTGGGTGAAATG-3″ (forward) and 5′-CCACGCCTACCAGGAAATCTC-3″ (reverse); IL-1β 5′-TCAGGCAGGCAGTATCACTCATT-3″ (forward) and 5′-GGAAGGTCCACGGGAAAGA-3″ (reverse); MCP-1 5′-TTAAAAACCTGGATCGGAACCAA-3″ (forward) and 5′-GCATTAGCTTCAGATTTACGGGT-3″ (reverse); IL-10 5′-GCTCTTACTGACTGGCATGAG-3″ (forward) and 5′-CGCAGCTCTAGGAGCATGTG-3″ (reverse); IL-6 5′-TAGTCCTTCCTACCCCAATTTCC-3″ (forward) and 5′-TTGGTCCTTAGCCACTCCTTC-3″ (reverse); MUC2 5′-ATGCCCACCTCCTCAAAGAC-3″ (forward) and 5′-GTAGTTTCCGTTGGAACAGTGAA-3″ (reverse); MUC3 5′-GCCGTGAATTGTATGAACGGA-3″ (forward) and 5′-CGCAGTTGACCACGTTGACTA-3″ (reverse).

### Western-blot analysis.

Cell samples were collected, and the protein was extracted with 1× RIPA buffer (Solarbio, China) and phenylmethylsulfonyl fluoride (PMSF) (1:1,000). Colon samples were weighed and homogenized in 1× RIPA buffer (10 μl/μg tissue) with PMSF (1:1,000) using the tissue breaker machine. The mixtures were centrifuged at 4°C for 10 min at 15,000 × *g*, and the supernatant was collected. Protein concentration was measured with a bicinchoninic acid (BCA) protein assay kit (Solarbio). Briefly, proteins were separated by 12% SDS polyacrylamide gels and then transferred onto polyvinylidene difluoride (PVDF) membranes (Bio-Rad Laboratories). The membrane was blocked with 5% skimmed milk for 1 h and incubated with antibody NRLP3 (ab214185; Abcam) overnight at 4°C and then incubated with second antibodies labeled with horseradish peroxidase (HRP) at room temperature for 1 h. Bands were visualized using an ECL kit (Fdbio Science, China), and band intensities were normalized by the results of β-actin.

### Immunohistochemical analysis.

Immunohistochemistry was performed in human and murine colon sections in order to determine the NLRP3 expression level. The tissue samples were immersed in 4% paraformaldehyde for 24 h and then embedded in paraffin for immunohistochemistry analysis. The tissues were sectioned into slices and then were dried in a drying oven at 60°C for a half hour. Anti-NLRP3 antibody (GB11300; Servicebio, Wuhan, China) was detected using anti-rabbit secondary antibody for a half hour at room temperature. The tissue slices were counterstained using a DAB chromogenic reagent kit. Then the intensity of NLRP3 staining was analyzed using ImageJ Java.

### Statistical analysis.

Data were expressed as means ± standard deviation (SD) and were analyzed using unpaired Student’s *t* test or Mann-Whitney U test or one-way analysis of variance (ANOVA) test. A *P* value of <0.05 was considered statistically significant. Statistical analyses were performed using Prism 7 software (GraphPad Software, Inc., La Jolla, CA, USA) and SPSS version 19.0 for Windows (SPSS, Inc., Chicago, IL, USA).

### Data availability.

Publicly available data sets were analyzed in this study. All data sets analyzed for the current study are available in the Gene Expression Omnibus (GEO; https://www.ncbi.nlm.nih.gov/geo/) with the accessions numbers GSE48959 and GSE75214 and the GMrepo database with the corresponding queries (https://gmrepo.humangut.info/).
