# Supplementary material for: Akkermansia muciniphila Alleviates Dextran Sulfate Sodium (DSS)-Induced Acute Colitis by NLRP3 Activation
Source: Microbiol Spectr. 2021 Oct 6;9(2):e00730-21. doi: 10.1128/Spectrum.00730-21 (PMC8510245; doi:10.1128/Spectrum.00730-21)
Supplement: Supplemental file 1 — Supplemental material. Download SPECTRUM00730-21_Supp_1_seq1.pdf, PDF file, 0.2 MB [file spectrum00730-21_supp_1_seq1.pdf]

Supplementary materials

A

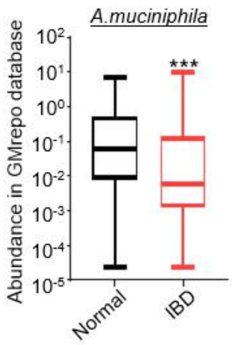

B

Bacteria abundance in GMrepo database

Bacteria abundance	Normal（Median）	UC（Median）
L. lactis	0.008296, n=1000	0.0129***, n=480
L. mucosae	0.00845, n=549	0.01256, n=43
B. adolescentis	0.4118, n=1000	0.3634*, n=473
B. breve	2.106, n=1000	0.01718***, n=317
B. longum	0.298, n=1000	0.1554***, n=658
S. salivarius	0.054, n=1000	0.08861***, n=1000
F. prausnitzii	2.458, n=1000	2.314, n=1000
A. muciniphila	0.593, n=1000	0.04162***, n=420

Supplementary Figure 1. (A)The level of *A. muciniphila* in the stool of normal people (n=1000) and IBD patients (n=399) in GMrepo database. (B)Details of the abundance of *L. lactis*, *L. mucosae*, *B. adolescentis*, *B. breve*, *B. longum, S. salivarius*, *F. prausnitzii* and *A. muciniphila* in the stool of normal people and UC patients in GMrepo database including exact sample numbers, medians and values, the graph (in Figure 1) exactly matched with this table. *p <0.05, ***p <0.001 (Mann Whitney test). IBD, inflammatory bowel disease; UC, ulcerative colitis.

Supplementary Table1. The clinical characteristics of UC patients

Cohort	Mean±SD	
	Low-*Akk* (n=6)	High-*Akk* (n=6)
Age	48±10.8	39.5±12.1
Sex		
Male	2	3
Female	4	3
Height（m）	1.7±0.1	1.6±0.1
Body weight(kg)	60.2±10.7	58.3±5.6
BMI(kg/m²)	21.7±2.4	21.8±1.9
Smoking		
Yes	0	0
No	6	6
Drinking		
Yes	0	0
No	6	6
Activity		
Mild	3	3
Moderate	3	2
Severe	0	1
